# Quality of sleep in patients with schizophrenia is associated with quality of life and coping

**DOI:** 10.1186/1471-244X-5-13

**Published:** 2005-03-03

**Authors:** John R Hofstetter, Paul H Lysaker, Aimee R Mayeda

**Affiliations:** 1 Department of Psychiatry, R.L. Roudebush VA Medical Center, 1481 W. 10^th ^St, Indianapolis, IN, 46202, USA and Department of Psychiatry, Indiana University School of Medicine, 1111 W. 10^th ^St, Indianapolis, IN, 46202, USA

## Abstract

**Background:**

While sleep disturbance is widespread in schizophrenia it is less clear whether sleep disturbance is uniquely related to impaired coping and perceived quality of life.

**Methods:**

We simultaneously assessed sleep quality, symptoms, and coping in 29 persons with schizophrenia or schizoaffective disorder in a post acute phase of illness. Assessment instruments included the Pittsburgh Sleep Quality Index; the Positive and Negative Symptom Scale; the Heinrichs Quality of Life Scale; and the Ways of Coping Scale. Multiple regressions were performed predicting quality of life and coping from sleep quality controlling for age and symptom severity. On a subset of seven subjects non-dominant wrist actigraphy was used as an objective check of their self-reported poor sleep.

**Results:**

Analyses revealed that poor sleep quality predicted low quality of life (r = -0.493; p = .022) and reduced preference for employing positive reappraisal when facing a stressor (r = -0.0594; p = 0.0012). Actigraphy confirmed poor sleep quality in a subset of subjects. They had shorter sleep duration (p < .0005), shorter average sleep episodes (p < .005) and more episodes of long awakening (p < 0.05) than community norms.

**Conclusion:**

The results are consistent with the hypotheses that poor sleep may play a unique role in sustaining poor quality of life and impaired coping in patients with schizophrenia. These associations may hold for community controls as well.

## Background

Many persons with schizophrenia report chronically disturbed sleep [1,2]. Independent of the phase of illness, sleep disturbance documented by polysomnography include: difficulties falling asleep, awakening too early and being unable to go back to sleep, a preference for being awake during the evening, reduced deep or slow-wave sleep (the most restorative stage of sleep), and short REM latencies [3-6]. In addition to being a source of distress, various forms of sleep disturbance have also been linked to heightened levels of thought disorder [7] and symptoms of excitement [8] and may portend relapse [9].

While sleep disturbance appears widespread in schizophrenia and is related to clinical features, less clear is whether it is also related to impaired coping that characterizes the disorder. Is poor sleep quality another factor that uniquely contributes to the difficulties persons with schizophrenia experience coping with stressors, and sustaining relationships and work? There appear to be several reasons to hypothesize that it may. Impaired sleep may make it difficult for persons with schizophrenia to cope with stressors [10]. Persons with schizophrenia and impaired sleep might feel, for instance, especially exhausted and highly inclined to avoid stressors and have difficulty seeing the positive aspects of daily challenges. Consistent with this, at least one study finds that damaged sleep quality in schizophrenia relates to longer periods of time spent in bed [2]. With low energy level and need for more time in bed, it seems a matter of intuition that persons might have great difficulty sustaining interpersonal relationships and adapting to the demands of a work setting. Additionally, beyond its effects on coping, we might expect persons with impoverished sleep, irrespective of their symptom level, to have less social and vocational satisfaction than those with better sleep. Indeed, one study indicates that sleep quality is associated with quality of life for patients with schizophrenia [11].

To examine relationships between sleep quality, coping and quality of life, we simultaneously assessed these domains along with symptom level in a group of patients with chronic schizophrenia and schizoffective disorder in a post acute phase of illness. To assess sleep quality we used the Pittsburgh Sleep Quality Index (PSI [12]), a standardized self-assessment tool that gauges sleep quality during the past month. We chose the PSI because it has been successfully used in several studies of persons with schizophrenia [2,11,13,14]. To assess coping we used the Ways of Coping Questionnaire (WCQ), a widely used instrument that measures the relative degree of preference for a number of coping strategies and which is sensitive to the maladaptive coping preferences common to schizophrenia [15]. To assess symptoms and quality of life, we used two instruments that are the gold standard in schizophrenia research: the Positive and Negative Syndrome Scale (PANSS [16]) and the Quality of Life Scale (QLS [17]). We predicted that, controlling for levels of positive and negative symptoms, poorer sleep quality would predict poorer quality of life overall, a greater preference for avoidant coping, and a lesser preference for coping by positive reappraisal.

Some doubt the abilities of persons with schizophrenia to report their own sleep patterns accurately. However, a study comparing polysomnography with subjective measures of sleep found that they were highly correlated in a group of patients with chronic schizophrenia [18]. Similarly, others found that persons with severe mental illness can generally report the quality of their lives as well as those with other non-psychiatric illnesses [19,20].

In addition, we wanted to know if actigraphy is an effective way of demonstrating sleep problems in the post-acute phase of schizophrenia. To address this question, we used actigraphy to monitor sleep patterns in a subset of this patient population. Wrist actigraphy consists of monitoring locomotor activity with a motion sensor slightly bigger than a wrist-watch that is worn on the non-dominant wrist. It is a common method used to assess sleep [21]. Actigraphy is valid and reliable for evaluating sleep patterns in insomnia, in diagnosing circadian rhythm disorders, and in assessing sleep in subjects who are unlikely to tolerate polysomnography [22]. There are small studies that suggest the feasibility of using actigraphy in assessments of sleep disturbance in patients with schizophrenia [23,24].

## Methods

### Participants

All subjects met the Structured Clinical Interview for DSM IV (SCID [25]) diagnosis DSM-IV-TR [26] criteria for schizophrenia or schizoaffective disorder. All were in the post acute phase of illness; with no hospitalizations or medication changes for at least one month. Most were patients with chronic schizophrenia who were in a VA day treatment setting. All patient-subjects participated in an informed consent procedure and signed informed consent forms for this research. A tabulated summary of the participants' characteristics is in Table 1.

**Table 1:** Subject symptoms and demographics

Subject Characteristics	Mean (S.D.)
Subjects Enrolled	29
Men	27
Women	2
Age	48 (7)
Education	12 (2)
Age at first hospitalization	25 (9)
Number of hospitalizations	12 (6)
SCID diagnosis	
Schizophrenia	23
Schizoaffective disorder	6
PANSS components	
Positive	19 (5)
Negative	19 (6)
Cognitive	19 (5)
Emotional	13 (4)
Excitement/Hostility	9 (3)
Medication (CPZ equiv.)	755 (752)

### Instruments

#### Actigraphy

Sensors (Model # 24.000, Ambulatory Monitoring Inc, Ardsley, NY) slightly bigger than a wristwatch were worn by subjects on their non-dominant wrists for 21 days. Actigraphic logs of the non-dominant arm have a high correlation with gross locomotor activity [27]. Actigraphy is validated and reliable for evaluating sleep patterns [22]. The data from each actigraph was processed by the software included with the actigraphic monitors.

#### Pittsburgh Sleep Quality Index (PSI)

The PSI is an effective instrument used to measure the quality and patterns of sleep. It differentiates "poor" from "good" sleep by measuring seven subscales: Subjective Sleep Quality, Sleep Latency, Sleep Duration, Habitual Sleep Efficiency, Sleep Disturbances, Use of Sleeping Medication, and Daytime Dysfunction over the last month. The client self-rates each of these seven areas of sleep by answering nine questions. Scoring of answers is based on a zero to three scale, and a score of three reflects the negative extreme on the Likert Scale. A global sum of "5" or greater indicates a "poor" sleeper. The PSI has internal consistency and a reliability coefficient (Cronbach's alpha) of 0.83 for its seven components. Numerous studies using the PSI have supported high validity and reliability.

#### Positive and Negative Syndrome Scale (PANSS)

The PANSS is a 30 item rating scale completed by clinically-trained research staff at the conclusion of chart review and a semi-structured interview. For the purposes of this study the five PANSS factor analytically derived components are used: Positive, Negative, Cognitive, Excitement and Emotional discomfort [28].

#### Quality of Life Scale (QLS [29])

The tool is a 21 item scale completed by clinically trained staff after a semi-structured interview and chart review that assesses quality of life. Items are scored on a 7-point scale with higher ratings representing higher levels of satisfaction. Items tap a range of essential aspects of psychosocial interactions incorporating four subscales: Interpsychic Foundations; Occupational Functions; Commonplace Objects; and Activities and Interpersonal Relationships. For the purposes of this study we used the sum of all items as an index of overall quality of life. Interrater reliability for this instrument has been reported to range between 0.85 and 0.93 [15].

#### Ways of Coping Questionnaire (WCQ [30])

The WCQ is a self-report instrument that asks participants to call to mind a recent stressor and then rate how often they have used 66 different behaviours to cope with that stressor. Scale scores are additively derived from individual items and divided by a total score to provide a relative score. This relative score reflects participants' relative preferences among a set of discrete coping strategies. Relative scores are generally preferable because, among other things, they control for response bias. For the purposes of this study we calculated the relative scores for two subscales: "Escape Avoidance," and "Positive Reappraisal." Escape Avoidance describes wishful thinking and behavioural efforts to actively escape or avoid the problem and includes items such as: "I refused to believe it had happened" and "I wished the situation would go away or be over with." Positive Reappraisal describes efforts to create positive meaning by focusing on personal growth and includes items such as: "I changed or grew as a person in a good way" and "I found new faith." Internal consistency assessed using Cronbach's alpha have been reported to range from 0.61 to 0.79.

### Procedures

Following informed consent participants were administered the PSI. PANSS, QLS, and WCQ were completed within the previous month for another study of the effects of personality on function in schizophrenia. Diagnosis was determined using the SCID. Ratings of sleep quality were elicited by a research assistant blind to the results of the PANSS, QLS and WCQ. We fitted a subset of patients with actigraphs that they wore for 21 days.

### Statistical analysis

The statistical analyses were Pearson correlations and Mixed Models analyses in SAS^® ^software. We also repeated the correlations for patients with schizophrenia separate from the patients with schizoaffective disorder.

## Results

Mean scores ± SD on the tests were: Quality of Life total 51.73 ± 16.16; Pittsburgh Sleep Quality Index 11.6 ± 4.57; Ways of Coping Questionnaire subscales: "Escape Avoidance," 0.138 ± 0.062 and "Positive Reappraisal" 0.125 ± 0.079. There was no correlation between QLS and "Escape Avoidance" scores, but a modest positive correlation between QLS and "Positive Reappraisal" (r = 0.421, p = 0.021).

To examine the associations between sleep and quality of life we next performed partial correlations, using the PSI total score to predict QLS total score and QLS subscores with age and positive and negative component scores as covariates. Similarly, we used PSI subscores to predict QLS total score. Results revealed that poor sleep was related to poor quality of life (total PSI vs total QLS, r = -0.493; p = .022). The PSI accounted for 24% of the variance in QLS. Total PSI did not correlate with any of the QLS subscores. The PSI subscores: "Subjective Sleep Quality", "Sleep Duration", and "Sleep Disturbances" correlated with total QLS at p < 0.05 but none were significant after making the Bonferroni correction for multiple comparisons.

To examine the associations between sleep and coping style we next performed partial correlations, using the PSI total score to predict WCQ Escape Avoidance, and WCQ Positive Reappraisal score with age and positive and negative component scores as covariates. Results revealed that poor sleep was related to a reduced preference for Positive Reappraisal (r = -0.594; p = 0.0012). Sleep quality was found to be unrelated to Escape Avoidance. The PSI accounted for over 37 % of the variance in coping.

Patients with schizoaffective disorder had worse sleep as measured by the PSI than did patients with schizophrenia (13.7 ± 4.4 versus 9.7 ± 4.7; t-test, p = 0.03). The two groups did not differ in total scores of the QLS or WCQ. The correlations between total PSI and total QLS, and between total PSI and Positive Reappraisal for patients with schizophrenia were significant (r = -0.516; p = 0.039 and r = -0.645; p = 0.006 respectively). The same correlations were not significant for patients with schizoaffective disorder.

Seven participants with schizophrenia accepted the activity monitors and were willing to wear them continually for up to 3 weeks. Seven patients completed actigraphic monitoring, and the actigraphic results are in Table 2. Actigraphy verified that participants with schizophrenia had less overall sleep and more interrupted sleep than published community norms.

**Table 2:** Sleep measures for subject subset and controls*

**Sleep Measure Means**	**Patients**	**Controls**	**Units**
Participants	7	>200	
Sleep Duration	1359 ^c^	1440	min/d
Sleep Proportion	25	33	% of d
# of Long Awake Episodes	10.2 ^a^	6.7	ea d
Mean Sleep Episode	28 ^b^	60	min
Longest Sleep Episode	105 ^c^	231	min

*Controls data provided by Ambulatory Monitoring

^a ^= p < .05; ^b ^= p < .005; ^c ^= p < .0005

## Discussion

The results are consistent with the hypothesis that poor quality sleep that typically characterizes schizophrenia may have a powerful impact on both patients' perception of their quality of life and their ability to cope with stress. Independent of age and symptoms, poorer sleep quality predicted poorer quality of life and greater difficulties appraising stressors in a positive light. This may suggest that among the deficits accompanying schizophrenia, poor sleep is often underappreciated. Impaired sleep may erode the ability of schizophrenic patients to cope with the routine stress associated with work and interpersonal relationships. Furthermore, chronic sleep deprivation may contribute to anergy that impairs attendance and work performance. Since the found associations are independent of symptom level they may be trait variables.

These findings are consistent with polysomnographic studies of sleep in schizophrenia which show that many sleep deficits that are not dependent on the acuity of illness [3-7]. The findings are also consistent with a prior study of sleep quality and quality of life in patient with schizophrenia [11]. We had an insufficient number of participants to confirm a correlation between PSI subscores and total Quality of Life.

The negative relationship between complaints of poor sleep quality and Preference for coping by Positive Reappraisal remained significant when the confounding effects of symptom acuity and age were partialled from the correlation matrix. One interpretation is that chronically disturbed sleep may erode both the ability to find positive meaning and the desire to achieve personal growth. Given the correlative nature of the data analysis, however, it is not possible to infer causality. It may be that poor coping or poor quality of life lead to greater difficulties sleeping or that another unmeasured variable may account for the relationships. We plan future longitudinal studies that may uncover if sleep changes typically precede or follow changes in either coping or quality of life.

There are other limitations to this study; most participants were male and in their 40s. Further experiments will be needed which include females and males in earlier phases of illness. The association of disturbed sleep and both quality of life and coping may hold for community controls as well.

## Conclusion

These findings may have important clinical implications. If poor sleep quality is indeed a critical factor in quality of life and coping impairments in schizophrenia, clinicians will need to focus on and aggressively treat sleep problems. Specific treatments could include training in sleep hygiene with a focus on regular waking and sleep times, avoiding naps, morning bright light, evening melatonin, or other hypnotic agents. Improved sleep may lead to improved ability to cope with stress, and increased energy. These would improve the quality of life and coping in patients with schizophrenia.

## Competing interests

The author(s) declare that they have no competing interests.

## Authors' contributions

JH participated in the design and coordination of the study and performed the statistical analysis and helped to draft the manuscript. PL conceived of the study and participated in its design and helped to draft the manuscript. AM participated in the design of the study and helped to draft the manuscript.

## Pre-publication history

The pre-publication history for this paper can be accessed here:



## References

[B1] Doi Y, Minowa M, Uchiyama M, Okawa M, Kim K, Shibui K, Kamei Y (2000). Psychometric assessment of subjective sleep quality using the Japanese version of the Pittsburgh Sleep Quality Index (PSQI-J) in psychiatric disordered and control subjects. Psychiatry Res.

[B2] Royuela A, Macias JA, Gil-Verona JA, Pastou JF, Maniega MA, Alonso J, Roman JM, De Paz F, Barbosa M, Rami-Gonzalez L, Boget T (2002). Sleep in schizophrenia: a preliminary study using the Pittsburgh Sleep Quality Index. Neurobiology of Sleep-Wakefulness Cycle.

[B3] Keshavan MS, Reynolds CFIII, Miewald MJ, Montrose DM, Sweeney JA, Vasko RCJ, Kupfer DJ (1998). Delta sleep deficits in schizophrenia: evidence from automated analyses of sleep data. Arch Gen Psychiatry.

[B4] Lee JH, Woo JI, Meltzer HY (2001). Effects of clozapine on sleep measures and sleep-associated changes in growth hormone and cortisol in patients with schizophrenia. Psychiatry Res.

[B5] Tandon R, Shipley JE, Taylor S, Greden JF, Eiser A, DeQuardo J, Goodson J (1992). Electroencephalographic sleep abnormalities in schizophrenia. Relationship to positive/negative symptoms and prior neuroleptic treatment. Arch Gen Psychiatry.

[B6] Wetter TC, Lauer CJ, Gillich G, Pollmacher T (1996). The electroencephalographic sleep pattern in schizophrenic patients treated with clozapine or classical antipsychotic drugs. J Psychiatr Res.

[B7] Zarcone VP, Benson KL (1997). BPRS symptom factors and sleep variables in schizophrenia. Psychiatry Res.

[B8] Hofstetter JR, Mayeda AR, Happel CG, Lysaker PH (2003). Sleep and daily activity preferences in schizophrenia: Associations with neurocognition and symptoms. J Nerv Ment Dis.

[B9] Tan HY, Ang YG (2001). First-episode psychosis in the military: a comparative study of prodromal symptoms. Aust N Z J Psychiatry.

[B10] Morin CM, Rodrigue S, Ivers H (2003). Role of stress, arousal, and coping skills in primary insomnia. Psychosom Med.

[B11] Ritsner M, Kurs R, Ponizovsky A, Hadjez J (2004). Perceived quality of life in schizophrenia: relationships to sleep quality. Qual Life Res.

[B12] Buysse DJ, Reynolds CFIII, Monk TH, Berman SR, Kupfer DJ (1989). The Pittsburgh Sleep Quality Index: a new instrument for psychiatric practice and research. Psychiatry Res.

[B13] Lazarus RS, Folkman S (1984). Stress, appraisal and coping.

[B14] Yamashita H, Mori K, Nagao M, Okamoto Y, Morinobu S, Yamawaki S (2004). Effects of changing from typical to atypical antipsychotic drugs on subjective sleep quality in patients with schizophrenia in a Japanese population. J Clin Psychiatry.

[B15] Lysaker PH, Wilt MA, Plascak-Hallberg CD, Brenner CA, Clements CA (2003). Personality dimensions in schizophrenia: associations with symptoms and coping. J Nerv Ment Dis.

[B16] Kay SR, Fiszbein A, Opler LA (1987). The positive and negative syndrome scale (PANSS) for schizophrenia. Schizophr Bull.

[B17] Heinrichs DW, Hanlon TE, Carpenter WT (1984). The quality of life scale:  An instrument for assessing the schizophrenic deficit syndrome. Schizophrenia Bulletin.

[B18] Rotenberg VS, Indurski P, Kimhi R, Hadjez J, Gutman Y, Shamir E, Barak Y, Elizur A (2003). The relationship between objective sleep variables and subjective sleep estimation in schizophrenia. Int J Psych Clin.

[B19] Herrman H, Hawthorne G, Thomas R (2002). Quality of life assessment in people living with psychosis. Soc Psychiatry Psychiatr Epidemiol.

[B20] Khatri N, Romney DM, Pelletier G (2001). Validity of self-reports about quality of life among patients with schizophrenia. Psychiatr Serv.

[B21] Poyurovsky M, Nave R, Epstein R, Tzischinsky O, Schneidman M, Barnes TR, Weizman A, Lavie P (2000). Actigraphic monitoring (actigraphy) of circadian locomotor activity in schizophrenic patients with acute neuroleptic-induced akathisia. Eur Neuropsychopharmacol.

[B22] Ancoli-Israel S, Cole R, Alessi C, Chambers M, Moorcroft W, Pollak CP (2003). The role of actigraphy in the study of sleep and circadian rhythms. Sleep.

[B23] Wirz-Justice A, Haug HJ, Cajochen C (2001). Disturbed circadian rest-activity cycles in schizophrenia patients: an effect of drugs?. Schizophr Bull.

[B24] Wirz-Justice A, Cajochen C, Nussbaum P (1997). A schizophrenic patient with an arrhythmic circadian rest-activity cycle. Psychiatry Res.

[B25] Spitzer RL, Williams JB, Gibbon M, First MB (1992). The Structured Clinical Interview for DSM-III-R (SCID). I: History, rationale, and description. Arch Gen Psychiatry.

[B26] Association AP (2000). Diagnostic and Statistical Manual of Mental Disorders.

[B27] Haug HJ, Wirz-Justice A, Rossler W (2000). Actigraphy to measure day structure as a therapeutic variable in the treatment of schizophrenic patients. Acta Psychiatr Scand Suppl.

[B28] Bell MD, Lysaker PH, Beam-Goulet JL, Milstein RM, Lindenmayer JP (1994). Five-component model of schizophrenia: assessing the factorial invariance of the positive and negative syndrome scale. Psychiatry Res.

[B29] Heinrichs RW, Ruttan L, Zakzanis KK, Case D (1997). Parsing schizophrenia with neurocognitive tests: evidence of stability and validity. Brain Cogn.

[B30] Folkman S, Lazarus RS (1988). Ways of coping questionnaire manual.

